# Deep Learning-Based Event Classification of Mass Photometry Data for Optimal Mass Measurement at the Single-Molecule Level

**DOI:** 10.1021/acsnano.5c13074

**Published:** 2026-01-19

**Authors:** Kishwar Iqbal, Jan Christoph Thiele, Dominik Saman, Jack S. Peters, Stephen Thorpe, Samuel Tusk, Jack Bardzil, Justin L. P. Benesch, Philipp Kukura

**Affiliations:** † The Kavli Institute for Nanoscience Discovery, 6396 University of Oxford, Dorothy Crowfoot Hodgkin Building, South Parks Road, Oxford OX1 3QU, U.K.; ‡ Physical and Theoretical Chemistry Laboratory, Department of Chemistry, 6396 University of Oxford, South Parks Road, Oxford OX1 3QZ, U.K.

**Keywords:** mass photometry, resolving power, measurement feedback, spatiotemporal information, 3D convolutional neural network

## Abstract

Mass photometry (MP) is a powerful technique for studying biomolecular structures, interactions, and dynamics in solution. It detects and quantifies small reflectivity changes at a glass–water interface during protein (un)­binding, with signals typically averaged over 100 ms. However, particle motion at the point of single-molecule measurement can compromise key metrics such as mass resolution, sensitivity, and concentration. We present a three-dimensional convolutional residual network trained via supervised learning to classify landing events based on their spatiotemporal dynamics. By analyzing 3D event thumbnails, our method isolates optimal single-molecule measurements, eliminating cumulative histogram artifacts and improving resolving power by up to a factor of 2. Validated across diverse experimental data sets, including resolved and partially resolved samples, and varying masses, concentrations, and integration times, our approach delivers robust performance under (sub)­optimal conditions. Our approach also provides measurement-level data-driven feedback, facilitating high quality MP measurements in challenging scenarios.

## Graphical abstract

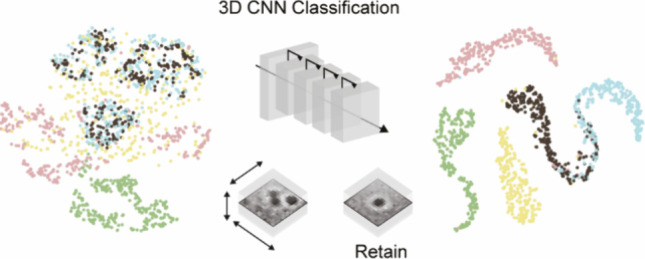

Mass photometry (MP)^1^ enables label-free studies of biomolecules with single-molecule sensitivity. By accurately measuring the mass of individual molecules and their complexes in solution, MP enables precise quantification of oligomerization, protein–protein, and protein–DNA interactions, as well as complex assembly.^2–10^ Typically, a sample is added to a microscope coverslip (Figure 1A), where nonspecific binding to the glass occurs. Because the refractive index of biomolecules (e.g., proteins, ∼1.46) differs from that of water (1.33), each binding event produces a local change in reflectivity. This change scales with molecular polarizability, which depends on the refractive index difference between the biomolecule and its surroundings, and is proportional to its volume. The similarity in optical properties and density among biomolecules results in a direct proportionality between polarizability and mass, enabling precise mass measurements at the single molecule level, given sufficient measurement performance.

**Figure 1:**
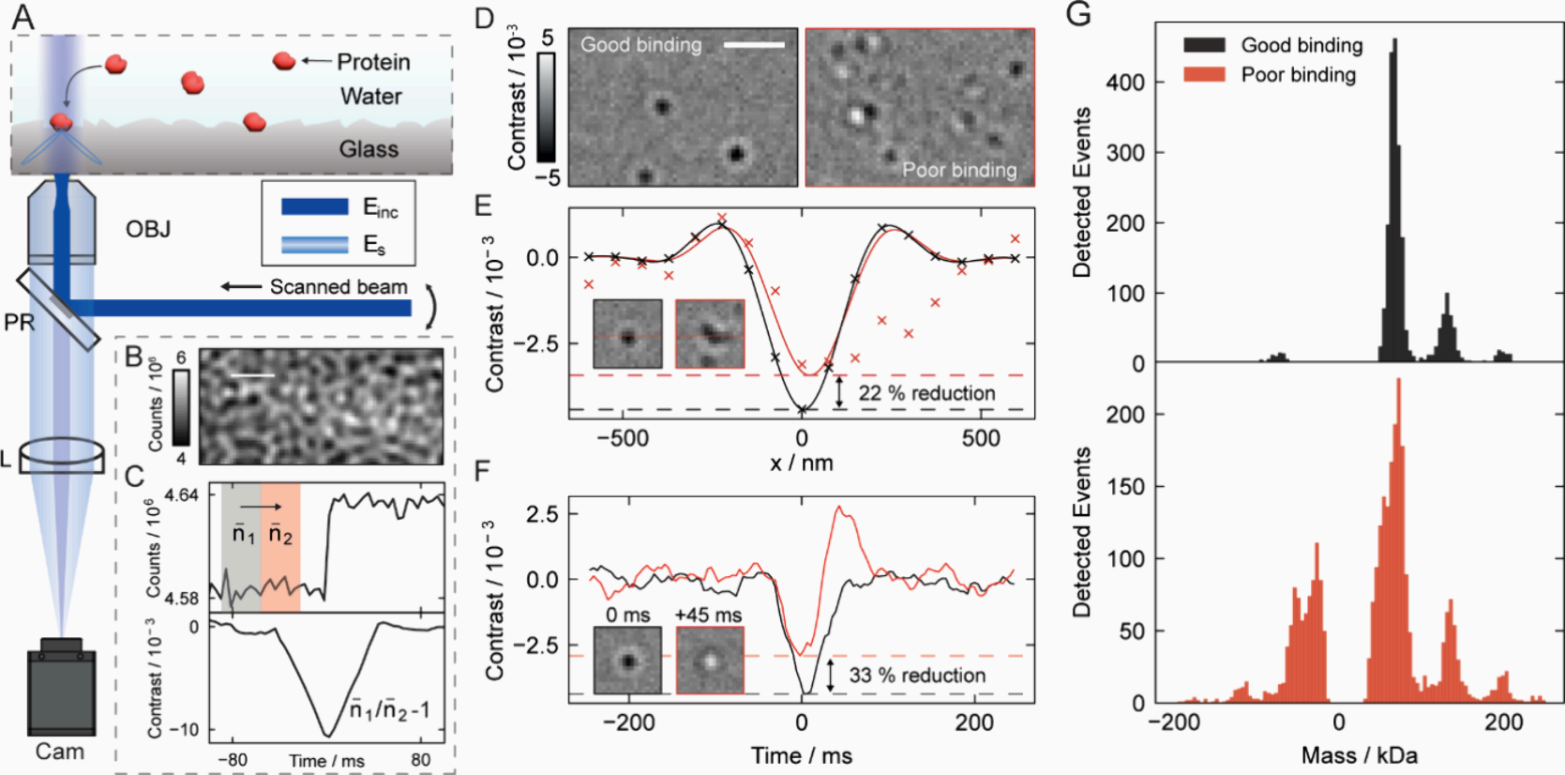
Mass photometry and challenges for accurate mass quantification. (A) Simplified schematic of a mass photometer and protein landing assay. (B) The raw camera image is dominated by scattering from the cover glass roughness. (C) Ratiometric image processing, achieved by dividing sequential frame substacks (*n*_1_ and *n*_2_), removes the background, revealing the weak signal from single-molecule landing events. (D) Images depicting (sub)­optimal binding of proteins to the glass surface. (E) Interference from overlapping molecules can lead to signal quantification inaccuracy (22% reduction shown). (F) Rapid unbinding within the ratiometric processing window can also result in a signal reduction (33%). (G) The cumulative impact of many inaccurately quantified events can introduce artifacts in the mass histogram, as shown in measurements of fresh BSA stock (black) and old stock (red). Abbreviations: PR: partial reflector, OBJ: objective, L: lens, Cam: camera, E_inc_: incident field, and E_s_: scattered field. Scale bar: 1 μm.

When experimental and shot noise are sufficiently reduced, these reflectivity changes can be visualized in real time, even against a substantial static optical background (Figure 1B). In cases of irreversible binding, the signal appears as a step function (Figure 1C) which is traditionally processed through a ratiometric analysis (Figure 1C,D).^11,12^ To enhance precision, the recorded point spread function (PSF) is fitted to an analytical model (Figure 1E, line), yielding the contrast that, when compared to a calibration, yields the molecular mass. The precision of these single-molecule mass measurements depends on background noise, the shot noise contribution to which can be reduced by temporal averaging of frames recorded before and after binding (*n̅*_1_, *n̅*_2_). However, this averaging is only successful and representative if binding is irreversible and unperturbed. In the context of measurement precision, we define an *optimal binding event* as one that produces a single, stable landing time trace persisting throughout the integration window. For example, the binding of a second, neighboring molecule (<1 μm, < 100 ms) distorts the PSF, resulting in an inaccurate mass estimate (Figure 1E, red). Similarly, transient unbinding can cause interference between binding and unbinding signals within the integration window, leading to contrast underestimation and mass error (Figure 1F, red). Other perturbations such as lateral movement due to imperfect binding—whether simple translation (“rolling”) or erratic oscillations (“wobbling”)—further disrupt the PSF and thus quantification accuracy. Recording and quantifying all of these events generates a mass histogram (Figure 1G). However, these suboptimal events can distort the histogram features, causing loss of resolution through mass broadening, artificial low-mass peaks, artifacts at negative mass from unbinding, and increased baseline noise from poorly quantified low-abundance events (Figure 1G, red).

To quantify resolution in mass photometry, we adopt the term *resolving power*, defined analogously to mass spectrometry as *m*/Δ*m*,^13–15^ where *m* is the mass and Δ*m* the specified peak width. For isolated peaks, we take the full width at half-maximum (FWHM) to determine Δ*m*. In cases of substantial peak overlap, we use the valley definition, where Δ*m* corresponds to the mass difference between two peaks of similar height such that the valley between them drops to a specified percentage (e.g., 10%) of the smaller peak. Importantly, this definition captures a mass-dependent, quantitative measure of MP performance, allowing consistent comparisons across samples and experimental conditions. Advancing the resolving power is as crucial for mass measurements as improving resolution has been for structural biology.^16^ It determines whether molecular species in increasingly complex mixtures can be distinguished or remain unresolved, thus transforming the applicability of the technology.

MP has gained rapid adoption due to its ease of use and ability to quantify mixtures. Poor binding remains a challenge, because it impacts key performance metrics—resolving power, sensitivity, and concentration range—complicating data interpretation. Surface functionalization can mitigate this issue but often lengthens and complicates experimental protocols.^17,18^ If a measurement contains a sufficient number of optimal binding events, they can in principle be extracted through analytical techniques such as residual filtering. However, these methods are used on a case-by-case basis and often require expert knowledge, are time-consuming, and can become subjective.

The introduction of deep learning has revolutionized the field of data-driven analysis.^19–28^ It has been widely applied across various biophysical techniques, including fluorescence-based microscopy,^29–32^ mass spectrometry,^33,34^ and cryo-EM,^35–37^ and more recently, in interferometric scattering microscopy.^38,39^ These techniques have yet to be explored in the context of mass photometry performance—crucial to enable quantification of progressively more challenging biomolecular samples and mixtures.

In this work, we implement a three-dimensional convolutional residual network trained via supervised learning on a data set of simulated surface binding events over an experimentally acquired background. The model classifies each single-molecule binding event based on its local spatiotemporal features, represented as a 3D-thumbnail input, enabling the identification and retention of high-quality measurements. We evaluate our model across diverse and representative test conditions, including optimal and suboptimal binding measurements, varying mass and concentration levels, and different integration times—validated on over 100,000 experimental and 3 million simulated events. We demonstrate our model’s ability to refine experimental data by correcting the mass histogram and eliminating artifacts caused by inaccurate quantification. Notably, we show improvements in resolving power by up to a factor of 2. The most challenging test case was heat shock protein 27 (HSP27, bird), a poorly binding highly polydisperse protein. Our model substantially increased the resolution enabling clear separation of previously unresolved species, with validation through native mass spectrometry. Additionally, our approach introduces a new layer of quantitative feedback by revealing the distribution of optimal and suboptimal events within a given measurement. This feedback enables more consistent, high-quality MP measurements at the technique’s performance limits, helping to unlock more complex systems that remain challenging for MP.

## Results and Discussion

### Quantifying the Effect of Suboptimal Binding

We broadly classify MP landing events into five categories: ‘binders’ (optimal) and four suboptimal types—‘unbinders’, ‘neighbors’, ‘rollers’, and ‘wobblers’, as illustrated in Figure 2A. These classifications are guided by domain knowledge derived from extensive analysis of experimental data, capturing recurring behaviors that commonly affect mass accuracy. To assess the impact of suboptimal binding on single molecule mass measurement performance, we simulated landing events for a 180 kDa protein under varying conditions (Figure 2B), adjusting parameters such as binding duration, unbinding probability, and lateral velocity to capture different landing dynamics (Table 1 and Figure S1). Our analysis reveals that 85% of optimally binding events achieve a relative mass error of less than 5%, compared to only about 30% of suboptimal events. This inaccuracy distorts the mass histogram, leading to broadening of peaks in the mass distribution, with a direct impact on resolving power, and an increased noise floor (Figure S2). Analyzing fit residuals provides a means to distinguish spatially dissimilar events, such as neighboring or rolling events. However, it fails to differentiate transient behaviors, such as rapid unbinding or wobbling, which require temporal information. Residuals also lack interpretability, leaving users unaware of why certain events yield poor quantification.

**Table 1:** Simulation Parameters for Training Data by the Event Class^a^

	binder	unbinder	neighbors	roller	wobbler
duration/frames	∞	[10,5], normal	∞	[4,8), uniform	[2,15), uniform
unbinding probability	0	1	0	0.75	0.5
velocity/nm ms^–1^	0	0	0	[1.7,18.6), uniform	[8.5, 3.5], normal
neighboring events	0	0	[1,20), uniform	0	0

a Event masses were randomly generated between 30 and 800 kDa, with a bias toward the low mass range below 100 kDa.

**Figure 2:**
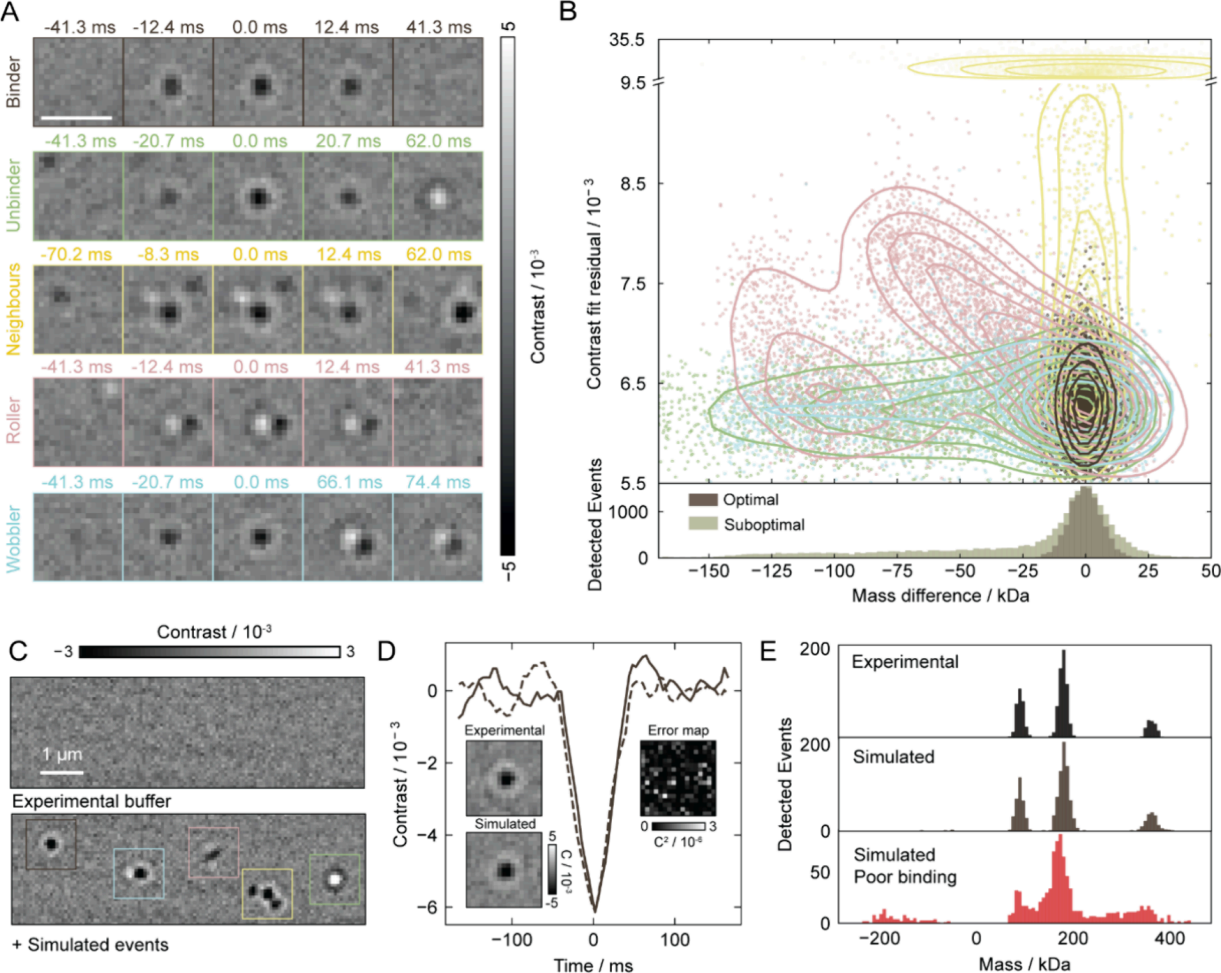
Analysis and simulation of the impact of suboptimal binding. (A) Five common landing event types in mass photometry. (B) Relationship between mass quantification accuracy and the fit residual for 25,000 simulated 180 kDa molecules. Optimal quantification is achieved for stably binding molecules, while all other event types increase inaccuracy in mass, primarily leading to underestimation. Counts are displayed below, with optimal event counts normalized for comparison. (C) Simulation framework using synthetic particle signals with diverse landing dynamics, overlaid on an experimental background. (D) Comparative analysis between an experimental (solid) and simulated (dashed) event, demonstrating high agreement, as seen in the error map. (E) Comparison between experimental and simulated mass histograms.

This analysis was enabled by our ability to accurately simulate MP data. We achieve this by modeling each binding event using experimentally determined PSFs superimposed on experimental background noise extracted from MP movies of buffer blanks without analyte (Figure 2C). To validate this approach, we compared experimental (solid) and simulated (dashed) events, demonstrating close agreement (Figure 2D). As a result, we can generate complete MP movies that produce mass histograms virtually indistinguishable from experimental data, both for optimal and suboptimal binding (Figure 2E). Notably, this simulation framework also serves as the foundation for our supervised learning training data set (see Methods for details).

### Deep Learning-Based Framework

We generated a data set of 25,000 simulated landing events, evenly distributed across event classes with varying parameters to capture a broad range of spatiotemporal characteristics (see Methods for details). As a first proof of principle of this framework, we simulated events spanning the 30–800 kDa mass range, with a skewed emphasis on the 30–100 kDa mass range, where the lower signal-to-noise (SNR) ratio presents greater challenges for accurate mass measurement. The data set was split 80:20 for training and validation to optimize model learning while ensuring reliable generalization (Figure 3A,B). We implemented a 50-layer 3D convolutional residual network based on the ResNet architecture (Figure 3C)^21,40^ to classify different types of landing events. Residual connections were integrated at the start of each residual layer, enabling deep feature extraction crucial for enabling separation between subtly wobbling events from optimally binding ones, particularly in low-SNR measurements (Figure 3D,E). The model receives the local spatiotemporal information on each landing event through the input of a (40 frames by 17 × 17 pixels) thumbnail, centered around the detected landing event. It then assigns class scores to determine the event type.

**Figure 3:**
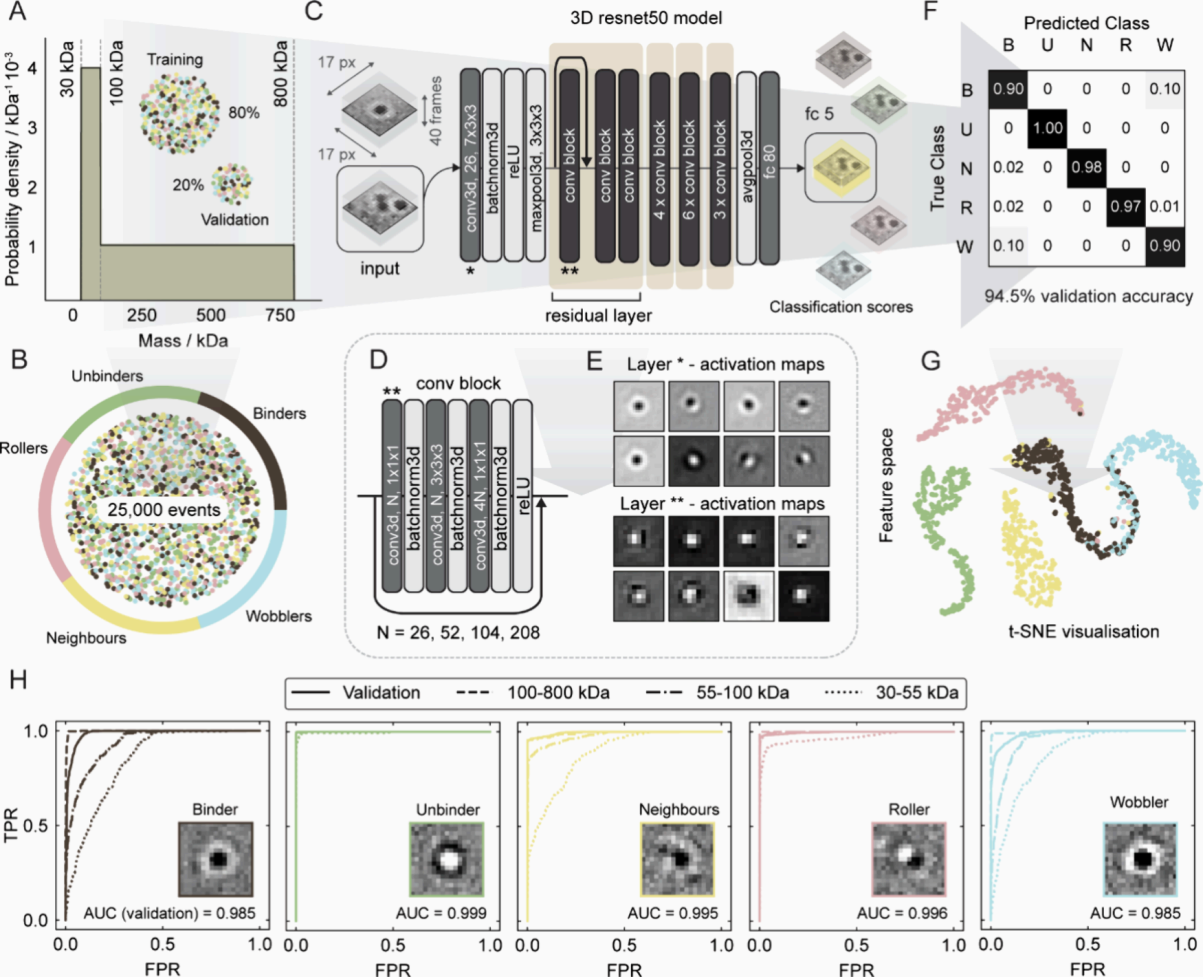
Deep learning-based event classification: an overview. (A) Mass distribution of the training and validation data set (80:20 split), with emphasis on the low signal-to-noise ratio range (30–100 kDa). (B) The data set was generated with a balanced distribution of 25,000 simulated events across event types. (C) A 50-layer 3D convolutional residual network classifies single-molecule landing events based on their local spatiotemporal dynamics, using a (17 px by 17 px by 40 frames) event thumbnail as the input. (D) The network consists of residual connections after the first convolutional block in each residual layer. (E) A temporal slice from the 3D activation maps illustrates the model’s response in the first two convolutional layers. (F) Confusion matrix from the validation data set, highlighting overall classification accuracy. (G) t-distributed Stochastic Neighbor Embedding (t-SNE) visualization of the event feature space, illustrating the separation between the different predicted classes. (H) ROC curves illustrating diagnostic performance by class on the validation data set, along with additional simulated data sets (1500 events each) segmented by the mass range. The model shows better performance for masses above 100 kDa.

After training (see Methods), our model achieved 94.5% validation accuracy. The confusion matrix revealed that binding and wobbling events were most frequently misclassified (Figure 3F), highlighting the ambiguity in separating events with very small perturbations. This trend was further reflected in the t-SNE visualization, which showed overlap between these two event types (Figure 3G). Comparison of raw and network feature spaces highlights the model’s ability to extract discriminative features and cluster events not separable in the unprocessed data (Figure S3). We further evaluated classification performance per class across the 30–55, 55–100, and 100–800 kDa mass ranges using receiver operating characteristic (ROC) curve analysis (Figure 3H). Table 2 presents the area under the curve (AUC) for each test case, demonstrating strong overall performance. Notably, high classification accuracy is maintained across a broad mass range, however, this becomes more challenging near the detection limit (30–55 kDa) due to the low SNR.

**Table 2:** Area Under the Curve (AUC) for the Simulated Validation Dataset (30–800 kDa, 5000 Events) and Three Additional Datasets Subdivided by the Mass Range (1500 Events Each), with Diagnostic Performance Separated by Class

	binder	unbinder	neighbors	roller	wobbler
validation	0.985	0.999	0.995	0.996	0.985
30 ≤ *m* (kDa) < 55	0.841	0.995	0.882	0.968	0.868
55 ≤ *m* (kDa) < 100	0.932	0.999	0.978	0.998	0.950
100 ≤ *m* (kDa) < 800	0.998	0.999	0.995	1.00	0.995

### Event Classification for Mass Photometry

We evaluated our model on experimental data with a variety of protein mass distributions and binding affinities to glass that are representative of the most often faced challenges for MP measurements (Figure 4). We retained only optimal binding events that strongly adhered to the glass to enhance MP performance. While in principle such selection could lead to an underrepresentation of weakly bound species, it is unlikely that a subset of protein complexes in a mixture would exhibit substantially lower binding affinity to glass, given the nonspecific nature of surface adsorption. Nevertheless, we caution users about the potential for selection bias when retaining only optimal binding events. In fact, an important aspect of our approach is that it provides feedback to the user about the nature of all binding events, enabling users to recognize and further investigate any excessive removal of specific subpopulations. An example could be DNA–protein complexes, which could result in lower surface adhesion due to the high negative charge of DNA. Performing our analysis on differently charged surfaces would reveal any bias in event selection. We set the binding score threshold based on the ROC curve analysis on the validation data, while balancing the need to retain sufficient counts per peak after filtering. We measured BSA as a reference protein, where both optimal and suboptimal binding are observed in independent measurements depending on sample quality (Figure 1). The model accurately classified events from the mixed BSA data set, as confirmed by inspection of the single-molecule measurements (Figure 4A). The suboptimal events were primarily responsible for counts appearing between BSA peaks due to erroneous mass quantification. These artifacts are often mirrored on the negative mass side of the histogram, as unbinding events. By selectively removing these poor events, the model effectively eliminates artifacts introduced by suboptimal landing. Importantly, the ratio between different oligomeric states is essentially unaffected. In cases where low-mass species may be underrepresented due to SNR-related misclassifications in mixed samples, this bias can be estimated and corrected (Figure S4).

**Figure 4:**
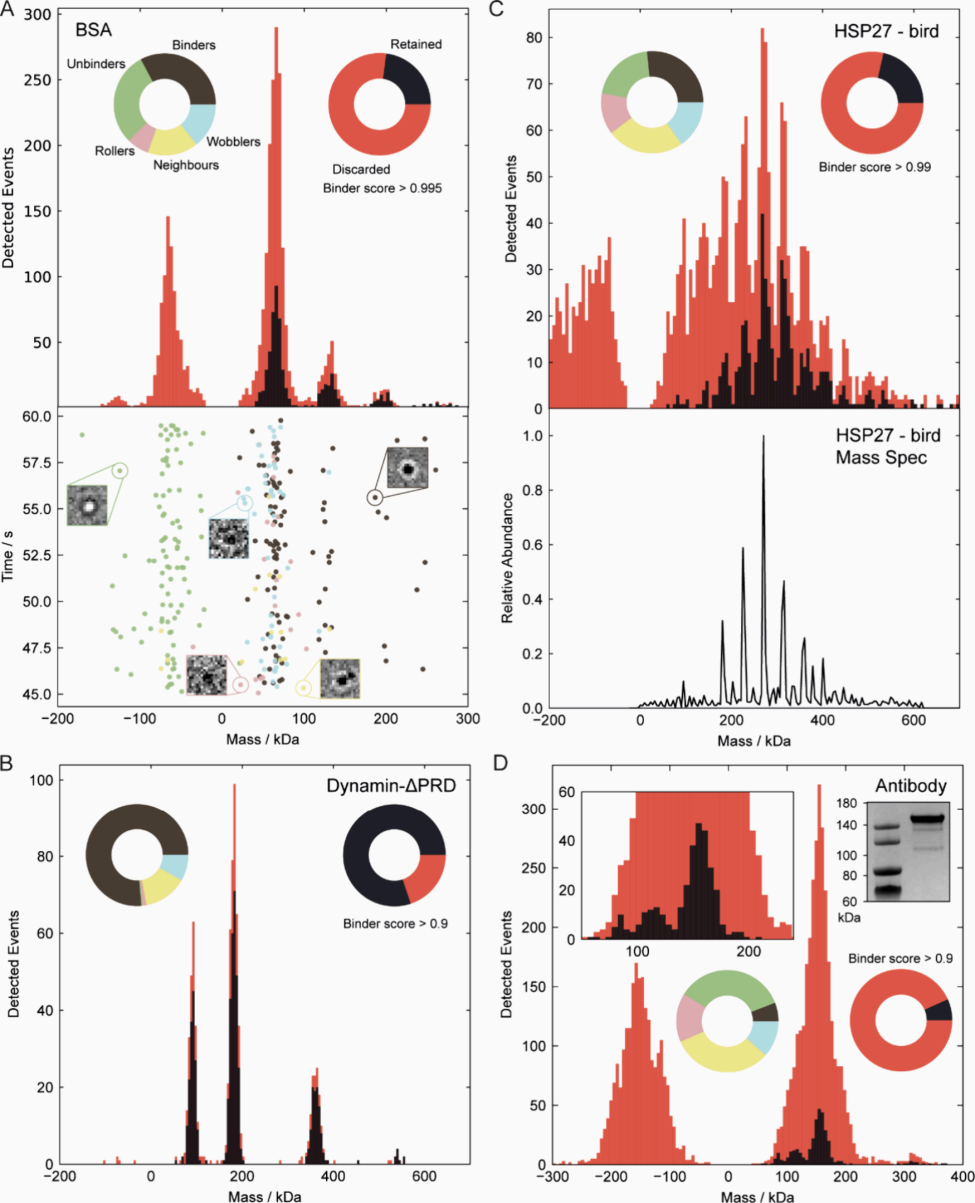
Identifying optimal single-molecule landing events for diverse test protein measurements. (A) Bovine serum albumin. Pie charts display the predicted event class distribution and selective retention based on the optimal binder score threshold. The mass histogram is shown before (red) and after (black) event filtering, with the distribution of events over time during the final 15 s of the landing assay shown below. (B) Dynamin-ΔPRD. (C) Heat shock protein 27 (HSP27). Mass distributions before and after filtering are shown (top), with validation provided by native mass spectrometry (bottom). (D) Anti-PSMC6 antibody, with validation provided by SDS-PAGE analysis (inset).

By contrast, dynamin-ΔPRD measurements represent a protein with high binding affinity to glass and exhibit predominantly optimal binding events. Here, the stochastic nature of landing events on glass and the event density led to neighboring events being the highest proportion of discarded events. This resulted in a largely unchanged mass distribution before and after filtering (Figure 4B). This represents the expected level of optimal performance, corresponding to a resolving power of approximately 10 for the 180 kDa dimer peak and ∼17 for the 360 kDa tetramer peak.

HSP27 represented the most challenging protein we tested, due to its broad mass distribution spanning much of the mass range over which our model was trained. It also presented many partially resolved peaks and exhibited poor surface affinity. Event classification and filtering revealed that the inclusion of suboptimal events (see Supplementary Video 1) not only elevated the baseline of the mass distribution but also artificially increased counts at the low-mass end. After their removal, a near-baseline resolved distribution was achieved and validated by native mass spectrometry, as shown in Figure 4C. We quantified the resolution improvement using the valley-to-peak ratio of the partially resolved peaks, as reported in Table 3. These were also compared to standard and user optimized results from the Discover^MP^ software (Refeyn). As DiscoverMP optimization requires some expertise, further details are provided in our MP protocol.^18^ Prior to filtering, the oligomeric peaks of HSP27 could not be resolved under the 30% valley criterion. Optimising the analysis parameters in Discover^MP^ allowed the 8–12-mer peaks to marginally meet this threshold. However, arbitrary adjustments to Discover^MP^ parameters can alter the mass distribution and lead to incorrect results, highlighting the need for expert tuning (Figure S5). Following our filtering approach, all peaks were resolved using the 30% criterion, with the 6–10-mer peaks even satisfying the more stringent 10% condition—demonstrating a substantial improvement in resolution. Residual analysis shows our method removes both high-residual outliers and low-residual intercluster events that broaden peaks and raise the baseline for HSP27 (Figure S6). Overlaying mass and residuals in t-SNE space highlights the largely mass-independent nature of our classification (Figure S7).

**Table 3:** Resolution of the HSP27 Bird Sample, Quantified by the Valley-to-Peak Ratio of Partially Resolved Peak Pairs^a^

	valley-to-peak ratio				
sample	DMP standard	DMP optimized	before event filtering	after event filtering	resolving power (30% valley)
6-mer, 8-mer (*m* = 4, *n* = 1271)	0.6 ± 0.1	0.5 ± 0.1	0.69 ± 0.05	0.06 ± 0.05	3.5
8-mer, 10-mer (*m* = 4, *n* = 1592)	0.38 ± 0.01	0.27 ± 0.05	0.42 ± 0.05	0.09 ± 0.01	4.5
10-mer, 12-mer (*m* = 4, *n* = 2057)	0.51 ± 0.03	0.30 ± 0.04	0.38 ± 0.03	0.25 ± 0.03	5.5
12-mer, 14-mer (*m* = 4, *n* = 2062)	0.41 ± 0.02	0.33 ± 0.03	0.38 ± 0.02	0.23 ± 0.04	6.5
14-mer, 16-mer (*m* = 4, *n* = 1710)	0.46 ± 0.03	0.43 ± 0.05	0.5 ± 0.1	0.3 ± 0.1	7.5

a Resolution was assessed using standard Discover^MP^ analysis settings (*n*_avg_ = 5, Threshold 1 = 1.20, Threshold 2 = 0.25), user-optimized Discover^MP^ settings (*n*_avg_ = 10, Threshold 1 = 2.60, Threshold 2 = 0.25), and custom Python analysis (*n*_avg_ = 10) before and after optimal event filtering. *m* indicates the number of independent repeat measurements, and *n* represents the estimated total number of events for each partially resolved peak pair. The mass of the HSP27 monomer is approximately 22.8 kDa.

For an anti-PSMC6 antibody sample, which exhibited poor surface affinity, the filtering process removed many events caused by significant unbinding from glass (Figure 4D). These events resulted in a mirrored negative mass peak which also increased the event density, leading to many neighboring events. Filtering substantially increased the resolving power of the 150 kDa peak from 3.9 ± 0.5 to 7.6 ± 0.3 across 8 independent measurements (29,447 total events), approaching the level expected for an optimal measurement (i.e., Figure 4B). The enhanced resolution revealed low-abundance degradation products in the antibody sample, observed as peaks below 120 kDa which were validated by SDS-PAGE analysis (Figure 4D, inset). We further tested our model for massference-p1 and apoferritin samples, where it effectively cleaned the mass histograms of artifacts from poorly quantified measurements (Figure S8).

In addition to suboptimal binding, excessive binding density can also contribute to inaccurate mass measurement. At low average event densities of 0.3 μm^–2^ s^–1^, we found negligible changes to the resolving power of the tetramer peak of dynamin-ΔPRD at 360 kDa before and after filtering (Figures 5A–C and S9 – experimental validation). Most events were retained due to the low probability of overlap and interference. As the event density increases with analyte concentration, the proportion of discarded events also increases due to a rise in overlapping events. As a result, the degree of improvement in mass resolution by our method increases with higher event densities. For example, at an event density of 34.6 μm^–2^ s^–1^, the resolving power improved from 5.3 ± 0.3 to 10.0 ± 1 after filtering. Despite the observed improvement, the mass resolution at higher concentration remains limited compared to that achievable at lower concentrations due to a general increase in imaging background.

**Figure 5:**
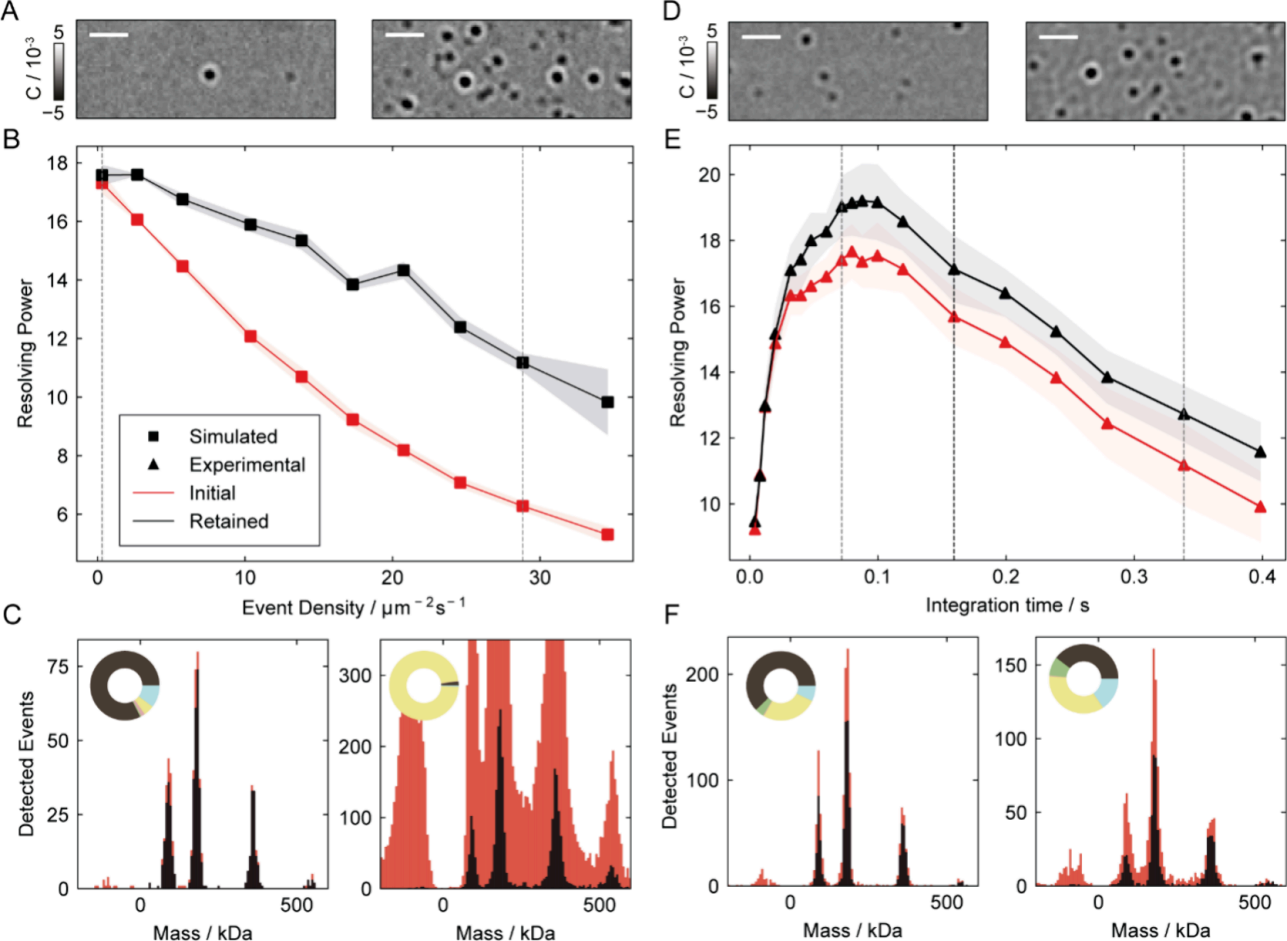
Optimal event selection improves resolution with increasing concentration and integration time. (Left) Effect of suboptimal event removal on simulated Dynamin-ΔPRD assays with increasing event density, with 5 repeats per concentration. (A) Frames for landing densities highlighted by the dashed lines in B. (B) Resolving power as a function of event density for the 360 kDa tetramer species, plotted before (red) and after (black) selective filtering. (C) Mass histograms corresponding to the highlighted densities. Binder score threshold was set to 0.8. (Right) Effect of event filtering on experimental Dynamin-ΔPRD assays analyzed across increasing integration times, repeated for 5 measurements. (D) Frames analyzed at 70 and 340 ms integration times, as highlighted by the dashed lines in E. (E) Resolving power as a function of integration time for the 360 kDa tetramer species, plotted before (red) and after (black) event filtering. The integration time was varied for the particle fitting process. Particle detection and classification were performed at two fixed integration times: 40 ms integration for data fitted below 160 ms, and 160 ms integration for data fitted above 160 ms. (F) Mass histograms corresponding to the highlighted integration times.

Increasing the integration time for a given analyte concentration also raises the probability of partially overlapping events. A longer temporal integration window also makes single-event measurements more susceptible to transient effects, such as rapid unbinding. We observe a clear improvement in resolving power of the tetramer peak of dynamin-ΔPRD as the integration time increases to 20 ms, attributed to the improving SNR of the measurements (Figure 5D–F). In this range, the filtered and nonfiltered data show minimal differences, owing to the short temporal window and low perceived event density. The increase in resolving power continues and reaches an maximal point between 80 and 120 ms. Within this range, we observe up to a 10% improvement, reaching 19 ± 1 following event filtering. This occurs due to the mitigation of the effect of overlapping/transient events. Beyond 120 ms, the mass resolution begins to decrease due to the increased density and incorporation of non-shot noise contributions. Although event filtering improves performance, it fails to achieve maximal levels. We partially attribute this to the model not being explicitly trained on experimental noise sources, such as those arising from the glass surface or fluctuations in the illumination. In principle, one could perform the training at different integration times to find overall optimal performance, which may vary depending on the sample of interest, for example shorter integration times for larger complexes. In practice, however, most MP experiments are performed at a set integration time (e.g., 40–100 ms) and training the model at a single integration time is sufficient.

We then evaluate the performance of our model near the quantitative detection limit at 40 ms integration with low-SNR measurements of protein A (42 kDa). For standard measurements, a low detection threshold required for low mass detection leads to detection of many false events, which manifests itself as a noise peak symmetric about zero mass in the mass histogram, illustrated on a buffer measurement (Figure 6A) and for protein A (Figure 6B, red). High detection thresholds, on the other hand, result in an asymmetric cut off on the low mass end of the detected peak (Figure 6B, inset (red)). After filtering protein A events using our model (Figure 6B, main panel and inset, black), we found a more symmetric peak centered around the expected mass of the protein (42 kDa), with false detections effectively removed. We applied a high classification threshold to retain only events with high certainty. In the 30–55 kDa simulated validation data, this corresponded to ∼61% classification precision and ∼23% recall for optimal events, illustrating the trade-off between retaining sufficient counts and improving detection quality. In practice, this resulted in discarding ∼70% of protein A events and ∼94% of all detected events. The threshold can be tuned: a more stringent cutoff increases precision at the expense of recall (for example, favorable when maximum mass resolution is desired), whereas a more permissive cutoff increases recall at the expense of precision (useful when discarding counts is less acceptable). This balance should be considered carefully for each sample, as in mixtures of high- and low-mass species, low-mass events can be disproportionately discarded and may require correction (Figure S4). Titration of protein A demonstrates that our approach remains sensitive enough to quantify changes in the protein A sample concentration without interference from spurious background detections (Figure 6C). While not representing a substantial improvement in low-mass detection, this analysis offers a useful benchmark of model performance near the detection limit and informs its application to smaller proteins discussed later. We validated the discarded events from the noise peak by evaluating them at a 140 ms integration time. The higher SNR shows that these spurious detections did not correspond to real events (Figure 6D). This becomes clear when comparing them to the retained events imaged at 140 ms integration (Figure 6E). While this analysis serves as a test case for our model, in practice, detection of low-mass species requires careful adjustment of integration time and detection filters, as these parameters directly affect sensitivity, resolution, and event density in MP. A more detailed discussion is provided in Kratochvíl et al.^18^

**Figure 6:**
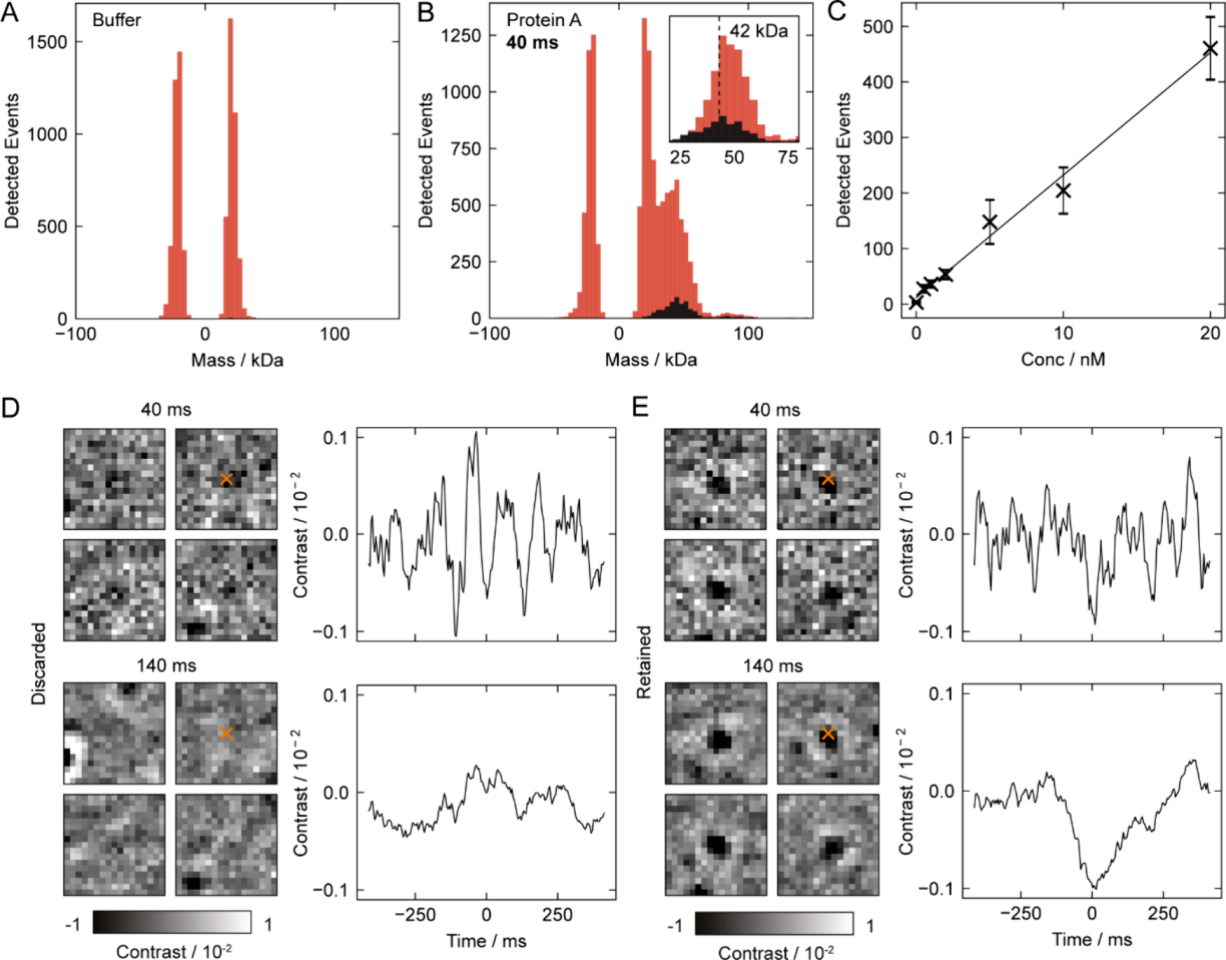
Detection of protein A (42 kDa) at 40 ms integration time. (A) Analysis of buffer blanks with low detection thresholds depicting false event detection. (B) Analysis of protein A under the same detection conditions. Selective event filtering was applied, with discarded events shown in red and retained events in black. The inset highlights the asymmetric peak (red) observed when applying high detection thresholds. (C) Titration of protein A counts. (D) Selected event thumbnails and time traces of discarded events, shown at 40 and 140 ms integration. (E) Selected event thumbnails and time traces of retained events, shown at 40 and 140 ms integration.

## Conclusions

Precise quantification of single-molecule landing signals underpins the mass measurement and resolving capabilities of mass photometry, enabling its broad applicability. Suboptimal surface binding and interference from neighboring molecules can distort signal quantification, limiting the sensitivity, resolving power, and concentration range of the technique. To address this, we adapted and optimized a 3D ResNet50 model to classify single-molecule landing events in MP based on their spatiotemporal features, enabling accurate separation and classification of optimal and suboptimal events. Applied across a diverse set of proteins varying in molecular mass, surface affinity, concentration, and integration time, our method removes artifacts and improves resolving power by up to a factor of 2, recovering near-optimal performance from substandard measurements. This improved robustness under nonideal conditions broadens the operational range of MP for experimentally challenging samples, while rejection of poor binding events improves the dynamic range important for detecting weak or low-affinity interactions. Additionally, the approach supports the development of automated, high-throughput pipelines by reducing reliance on expert intervention during analysis.

The application of machine learning (ML) to interference-based microscopy for nanoscopic imaging has primarily focused on low SNR particle detection to date.^38,39^ These studies tested the algorithms on experimental data on the order of ∼1000 particles. Our work shifts the emphasis toward enhancing resolving power, advancing the technique as a quantitative analytical tool, testing on over 100,000 experimental particles. A notable consequence of our approach is the interpretable feedback it provides on the distribution of event types within a given measurement. This emergent property enables users to make informed adjustments on concentration, binding conditions, or sample preparation, facilitating routine acquisition of high-quality data.^18^ Such a machine vision-based filtering approach also shows great promise for application in single-molecule tracking on lipid bilayers by MP,^41,42^ where extensive filtering of particles is required. In this first application, we discard suboptimal events to improve mass precision. In future applications, their spatiotemporal signatures could be leveraged through full 3D fitting of event thumbnails to extract diffusivity, transient behaviors, and corrected mass estimates, as well as by quantifying their relative abundance to diagnose surface properties or passivation quality. Despite this, MP detection and resolution remains limited by an uncharacterized, speckle-like dynamic background that emerges at high averaging.^43^ Without a clear understanding of this phenomenon, our ability to simulate and surpass the limits of MP using supervised learning will remain limited.

This work represents a significant step toward integrating deep learning into quantitative mass photometry. Owing to its lightweight, thumbnail-based architecture, our network trains rapidly and lends itself to transfer learning and adaptation across local, individual instruments. We invite the community to retrain and refine the model on their own data to accelerate broader adoption. Notably, our framework integrates with established particle detection pipelines, suggesting exciting opportunities for synergy with emerging ML-based methods that jointly enhance sensitivity and resolution. While the theoretical resolution limit of MP remains at ∼5 kDa FWHM under current optical constraints,^43^ our approach offers a practical path toward bridging the gap to current experimental performance (∼20 kDa). In combination with advances in detection, particle fitting, analytical corrections, and next-generation microscope designs,^44,45^ such developments promise to unlock the full potential of mass photometry as a robust, high-precision tool for advanced biomolecular quantification in solution.

## Methods

### Sample Preparation

Bovine serum albumin (BSA; lyophilized powder, Sigma-Aldrich), Anti-PSMC6 antibody [p42-23] (Abcam) and protein A from *Staphylococcus aureus* (lyophilized powder, Sigma-Aldrich) were diluted to 10–60 μM stock solutions in Dulbecco’s PBS. Dynamin-ΔPRD samples were prepared as detailed in Foley and Kushwah et al.^42^ The expression and purification of HSP27 (HspB1, bird) were performed as previously described.^46^

### Mass Photometry Measurement

All measurements followed the procedures outlined in the established protocol.^18^ Briefly, we performed all measurements using a Two^MP^ instrument (Refeyn) at room temperature. We cleaned No. 1.5 coverslips (50 × 24 mm, VWR) sequentially by 5 min sonication in milli-Q water, isopropanol, and milli-Q water, and dried using nitrogen. Grace Bio-Laboratories reusable CultureWell gaskets (3 mm diameter × 1 mm depth) contained the sample. We dispensed 4 μL of buffer to the gasket and focused the instrument with the autofocus function. We prepared working solutions by diluting samples to a final concentration of 1–100 nM and incubated for 2 min before measurement. Finally, we added a volume of 16 μL of the working solution, aspirated, and initiated measurement.

We recorded 60 s measurements with the small field of view setting (29.8 μm^2^) using the Acquire^MP^ software (Refeyn). Frame binning was set to 3, and data was acquired at 726 fps with an exposure time of 1.3 ms. Subsequent data analysis consisted of image preprocessing, particle picking, fitting, classification and mass histogram production. We performed these analyses using custom python scripts. Detailed information on particle classification is given below. Briefly, we performed particle picking by implementing pre-established algorithms and workflows.^18,47^ Unless stated otherwise, we conducted all analyses with an integration time of 40 ms (*n*_avg_ = 10). To quantify the event contrast, we fitted an experimental point spread function (ePSF) to the detected particles; details of its generation are provided below. For mass calibration, we measured Dynamin-ΔPRD alongside each experimental set. Using the calibrated mass values and fitted events, we constructed mass histograms to represent the distribution for each sample.

### Training Data Generation

We generated training data by simulating particles landing on an experimental background. An experimental point spread function (ePSF) modeled the landing particles. We derived the ePSF model from the event thumbnails of the dimer species (180 kDa) of Dynamin-ΔPRD (3,302 events from 5 independent measurements), selected due to its sufficient SNR and abundance. We interpolated and centered thumbnails on the central peak, retaining the top 80% of thumbnails with high similarity scores to average and generate a representative ePSF model for the instrument. While the underlying physical model of the interferometric point spread function (iPSF) is well understood,^48–50^ the imaging conditions used in MP where weak scatterers are imaged at the interface over a small field of view are well suited to the empirical model employed. We compared our ePSF model with a simple analytical PSF representation for MP, constructed from the superposition of two jinc functions multiplied by a Gaussian kernel.^42^ Both models yielded comparable performance in contrast estimation, however the ePSF achieved lower fit residuals for higher-mass species and was therefore selected for training data simulation. Further detail on the performance of contrast estimation and fundamental bounds has been described elsewhere.^43^

Simulated landings were added directly onto experimental background movies at the intensity level. Experimental validation confirmed that this simplification was sufficient for effective network training. Although this approach does not capture field-level neighbor interference or phase-sensitive distortions, it was adequate for training under the conditions tested, with potential misestimation expected only at very high event densities or for very large particles.

#### Optimal Event Simulation (Binders)

To simulate stable, optimal binding, we randomly generated a single landing event (*x*_*i*_, *y*_*i*_, *t*_*i*_) on top of a clean buffer movie and added it to all subsequent frames. We generated the mass of the event (30 ≤ *m*_*i*_ < 800 kDa) to form the distribution shown in Figure 3A. After ratiometrically processing the movie (*n*_avg_ = 10), we extracted a thumbnail (40 frames × 17 px × 17 px) centered around the event. We then discarded the simulated movie and repeated the process with a clean, randomly selected buffer for every simulated event.

#### Suboptimal Event Simulation

We introduced additional protein dynamics to simulate suboptimally binding events. By limiting the addition of the landing event for *t*_u_ frames, the simulation accounted for *unbinding events*. The value of *t*_u_, drawn from a normal distribution with a mean of 10 and a standard deviation of 5, truncated between 0 and 20 frames. To simulate *rolling events*, the model added a landing event that moved in a singular direction for *t*_r_ frames at a speed *v*_r_. The value of *t*_r_ came from a uniform distribution ranging from 4 to 8 frames, while the model drew *v*_r_ from a uniform distribution between 1.7 and 18.6 nm ms^–1^. Upon completing its motion, the rolling event either remained stationary or detached from the surface, with a 0.75 probability of unbinding. The model simulated *neighboring events* by introducing an optimal binder, followed by adding *N*_n_ other binders randomly positioned within the event thumbnail. The value of *N*_n_ ranged from 1 to 19 with equal probability. *Wobbling events* simulated proteins that followed a random walk, with its direction and speed (*v*_w_) updated at each frame over its wobbling duration (*t*_w_). The value of *t*_w_ was drawn from a uniform distribution between 2 and 15 frames, while *v*_w_ was sampled from a normal distribution with a mean of 8.5 nm ms^–1^ and a standard deviation of 3.4 nm ms^–1^. After wobbling, the protein either remained stationary or detached from the surface with a 0.5 probability. Table 1 summarizes all simulation parameters. Figure S1 depicts the effects of varying simulation parameters.

25,000 simulated events composed the training data set. We equally represented all event classes for balanced representation. Figure 3A displays the mass distribution of the training data, with a bias toward the low SNR regime (30 ≤ *m* < 100 kDa) to compensate for the increased prediction uncertainty in this range.

### Model Architecture

Figure 3C illustrates the 3D-ResNet based deep learning model^21,40^ used for classification. The model receives a 3D spatiotemporal thumbnail input, centered on the detected landing event, with dimensions of 40 frames × 17 pixels × 17 pixels. The model consists of an initial 3D convolutional layer (7 × 3 × 3) followed by batch normalization, a ReLU activation layer and max pooling (3 × 3 × 3). This is followed by 4 residual layers consisting of 3, 4, 6, and 3 convolutional blocks, respectively. Residual connections with identity downsampling were implemented after the first convolutional block in each residual layer. Each convolutional block consisted of three 3D convolutional layers followed by batch normalization and a final ReLU activation layer. After the final residual layer, global average pooling and two fully connected layers progressively reduce the feature dimensions to 5 in the final output layer.

### Model Training

Data preprocessing consisted of removing any thumbnails that contained NaN values. We performed standardization on a per-thumbnail basis using a *z*-score

$$Z_{\mathrm{in}}=\frac{\left(X_{\mathrm{in}}-\mu_{\mathrm{in}}\right)}{\sigma_{\mathrm{in}}}$$ 1

where *Z*_in_ represents the standardized thumbnail, *X*_in_ is the input thumbnail, and μ_in_ and σ_in_ are the mean and standard deviation of the input thumbnail. This standardization generalized mass bias during training and improved model performance across the entire mass range. We split the data set 80:20 for training and validation, with a batch size of 10 and random shuffling.

Model parameters were randomly initialized prior to training. We trained and optimized the model using a cross-entropy loss function and stochastic gradient descent at a learning rate of 0.1. A learning rate scheduler reduced the learning rate by a factor of 0.1 if the validation loss plateaued for 5 consecutive epochs. Gradients were clipped to a maximum norm value of 0.5 to prevent exploding gradients in deeper layers. Training occurred for 20 epochs. The validation accuracy plateaued at 94.5% after 15 epochs, at which point the model was accepted to avoid overfitting to the training data. This was performed in *Python* 3.8.8 using *PyTorch* 1.8.1 on a system with an Intel CPU (Family 6, Model 140), 15.8 GB RAM, and an NVIDIA GeForce MX450 GPU (2 GB VRAM). Training required approximately 64 min, indicating that the task can be completed with relatively modest computational resources.

The model was trained to evaluate thumbnails ratiometrically processed with an integration time of 40 ms (*n*_avg_ = 10). A second model was also trained, designed to perform on data processed with an integration time of 160 ms (*n*_avg_ = 40, corresponding to 80 frames × 17 px × 17 px thumbnails).

### Evaluation and Validation

#### Confusion Matrix

A confusion matrix was calculated from the validation data set and shown in Figure 3F.

#### Receiver Operating Characteristic (ROC) Curves

Figure 3H shows the ROC curves separated by event mass and class, with Table 2 reporting the AUC. We performed this analysis for the entire validation set of 5,000 events, covering a mass range from 30 to 800 kDa. To further assess performance across three specific mass ranges (30–55, 55–100, and 100–800 kDa), we simulated and evaluated 1500 additional events within each range, distributing them evenly across all event classes. We used the model as a binary classifier to assess the performance across each class. The output of the trained model was passed through a sigmoid function to obtain classification scores, and ROC curves were generated by varying the decision threshold for the target class.

True positive rate (TPR, also called recall) and false positive rate (FPR) are defined as

$$\mathrm{TPR}=\frac{\mathrm{TP}}{\mathrm{TP}+\mathrm{FN}},\;\mathrm{FPR}=\frac{\mathrm{FP}}{\mathrm{FP}+\mathrm{TN}}$$ 2

where TP, FN, FP and TN are the number of true positives, false negatives, false positives and true negatives predicted by the classifier.

We selected decision thresholds for binder events by optimizing the F1 score on validation data sets (Figure S10), providing a balanced trade-off between recall and precision (distinct from mass precision),

$$\mathrm{Precision}=\frac{\mathrm{TP}}{\mathrm{TP}+\mathrm{FP}},\;F1=2\frac{\mathrm{Precision}\times \mathrm{Recall}}{\mathrm{Precision}+\mathrm{Recall}}$$ 3

The optimal threshold can depend on sample mass and experimental goals, but as a general guideline we recommend starting at 0.9. Thresholds can then be increased to prioritise high-confidence events and maximum mass resolution (at the cost of discarding more counts) or decreased to retain more events.

#### Experimental Data Evaluation

We extracted and standardized 3D thumbnails (40 frames × 17 pixel × 17 pixel) centered on each detected event. The deep learning model assigned class scores to each thumbnail. We determined an optimal classification threshold for the binder class and retained only events with scores above this threshold. To assess the improvement in resolution performance, we determined the FWHM by fitting a Gaussian curve to clearly resolved mass peaks. For partially resolved peaks, such as those observed with HSP27 – bird, we calculated the valley-to-peak ratio (VPR) for each peak set by taking the ratio between the minimum point in the valley and the height of the smaller peak.

#### Varying Event Density

We evaluated the effect of event filtering on mass FWHM for increasingly dense movies using simulated dynamin-ΔPRD landing assays with optimal binding. We quantified the results based on the tetramer species (360 kDa). We simulated each density with 5 repeats. To specifically isolate the effects of event density, we simulated each movie with a constant oligomeric distribution. We applied a binder score threshold of 0.8 to retain the most optimal events, recognizing that most events had neighboring signals.

#### Varying Integration Time

We evaluated the effect of event filtering on mass FWHM for various integration times using dynamin-ΔPRD. We quantified the results based on the tetramer species (360 kDa). To perform classification, we trained two models at distinct integration times: 40 ms (*n*_avg_ = 10) and 160 ms (*n*_avg_ = 40). We then varied the integration time of the detected and classified events for subsequent analysis. For integration times below 160 ms, we used classifications from the first model; for times above 160 ms, we used classifications from the second model. This was repeated for 5 independent measurements.

#### Low Mass Proteins

To evaluate performance on low mass proteins, we tested the model on protein A. To prevent cutoff by the detection algorithm, we set permissive filter thresholds: Filter 1 = 0.01, Filter 2 = 0.15. We performed classification with a high binder score threshold of 0.997 to reduce the false positive rate in the low mass regime. After classification, we applied a nearest neighbor filter to remove multiple detections of the same landing event. This analysis was carried out across a titration series, with a minimum of four repeats per concentration.

#### Validation

We validated the mass distribution of the antibody measurements against SDS-PAGE analysis and the mass distribution of the HSP27 measurements using native mass spectrometry.^46^ We benchmark the performance on the HSP27 measurements against analysis from the Discover^MP^ software (v2024 R1) across various Filter 1 and Filter 2 settings, with results summarized in Table 3 and Figure S6. Additionally, classification outcomes were further evaluated by comparison to the fit residuals (*r*), which were calculated as the square root of the sum of squared residuals between the measured events *X*(*i*, *j*) and fitted events *X*_fit_(*i*, *j*), as illustrated in Figure S5,

$$r=\sqrt{\sum_{i=1}^{17}\sum_{j=1}^{17}(X(i,j)-X_{\mathrm{fit}}(i,j){)}^{2}}$$ 4

## Supplementary Material





## Data Availability

The data and code supporting the findings of this study are publicly available in the Oxford Research Archive (ORA) at https://ora.ox.ac.uk, under DOI 10.5287/ora-nbpqmexqv. The repository contains example datasets, trained deep learning models, analysis notebooks, and code sufficient to reproduce the main results reported in this Article, including experimental point spread function (PSF) generation, model training, and application to mass photometry data.

## Notes

The methods, results, and analyses performed in this study including simulations of binding dynamics to illustrate mass broadening, analytical corrections for classification errors, and comparative analyses against DiscoverMP filter settings; residual-distribution plots and t-SNE feature-space embeddings (main text) (PDF)
Event classification and filtering revealed the inclusion of suboptimal events (MOV)
