# Supplementary material for: Deep Learning-Based Event Classification of Mass Photometry Data for Optimal Mass Measurement at the Single-Molecule Level
Source: ACS Nano. 2026 Jan 19;20(4):3410–22. doi: 10.1021/acsnano.5c13074 (PMC12875028; doi:10.1021/acsnano.5c13074)
Supplement: Supplementary file 1 [file nn5c13074_si_001.pdf]

Deep learning-based event classification of mass photometry data for optimal mass measurement at the single-molecule level

Kishwar Iqbal[1,2], Jan Christoph Thiele[1,2], Dominik Saman[1,2], Jack S. Peters[1,2], Stephen Thorpe[1,2], Samuel Tusk[1,2], Jack Bardzil[1,2], Justin L.P. Benesch[1,2], Philipp Kukura[1,2]*

1) The Kavli Institute for Nanoscience Discovery, University of Oxford, Dorothy Crowfoot Hodgkin Building, South Parks Road, Oxford OX1 3QU, UK

2) Physical and Theoretical Chemistry Laboratory, Department of Chemistry, University of Oxford, South Parks Road, Oxford OX1 3QZ, UK

*Email: philipp.kukura@chem.ox.ac.uk

Supporting Information

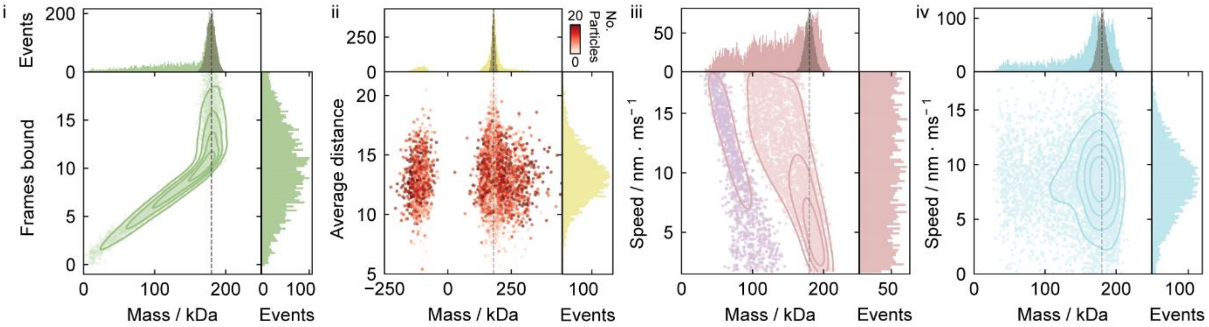

Fig S1 – The mass broadening effect of varying the simulated dynamics of suboptimal 180 kDa events. (i) Effect of transient unbinding as a function of frames bound. (ii) Effects of particle density and proximity in high event density thumbnails. (iii) Effect of rolling velocity. (iv) Effect of wobbling velocity.

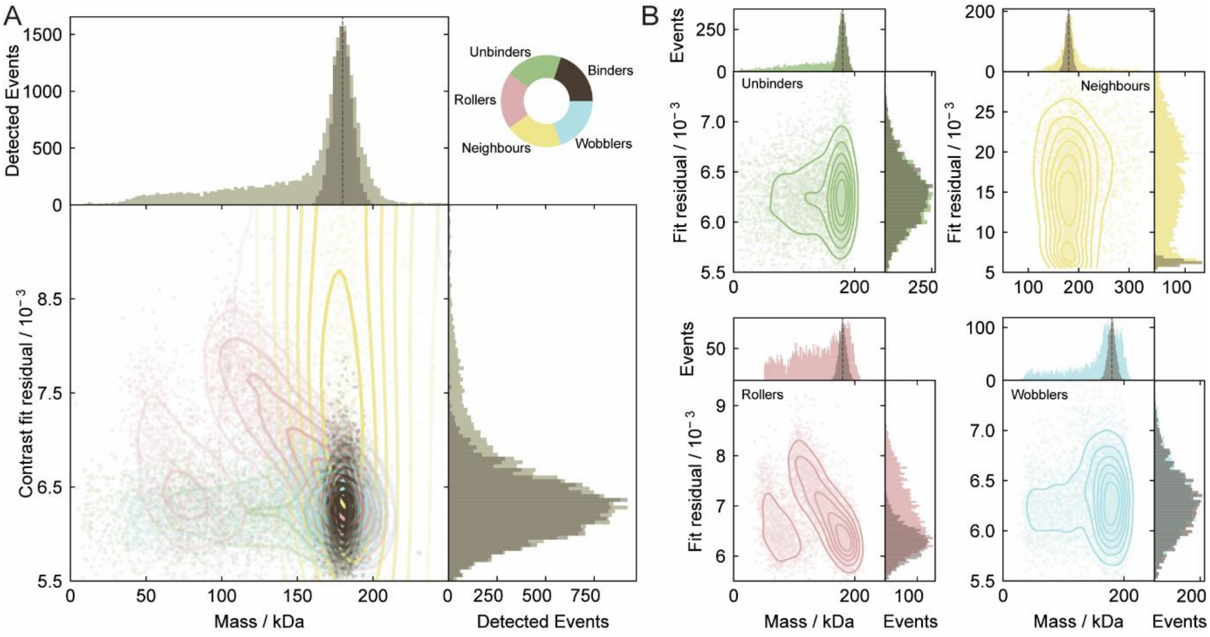

Fig S2 – Investigating the effect of suboptimal binding on mass photometry through simulation. (A) Contour plot depicting 25,000 simulated 180 kDa events, evenly distributed across each event class with different simulation parameters. Mass and residual histograms for all combined classes are shown (light), with the normalised histograms for the binder class overlaid (dark). Mass broadening is observed with a shoulder and increased base line noise extending across the low mass range. (B) Mass-residual contour plot separated by event class, demonstrating the mass broadening effect of transient behaviours such as rapid unbinding or wobbling, which cannot be distinguished using the 2D information in fit residuals alone.

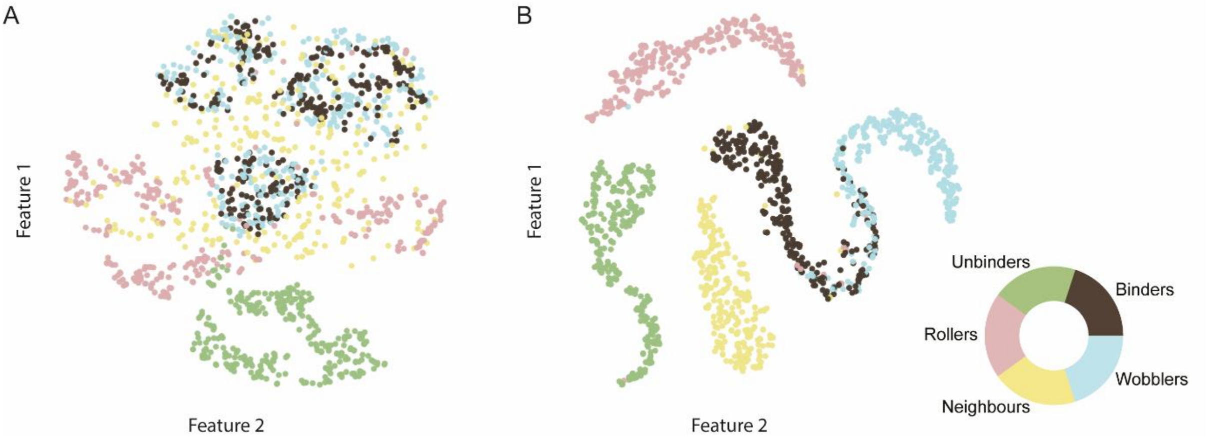

Fig S3 - Underlying structure in feature spaces. (A) t-SNE embedding of z-scored raw thumbnails shows only weak innate clusterability, with substantial overlap between event classes, particularly binders, wobblers and neighbours. (B) t-SNE embedding of the learned network features reveals clear separation of classes, demonstrating that the model extracts discriminative spatiotemporal features not readily apparent in the raw input data.

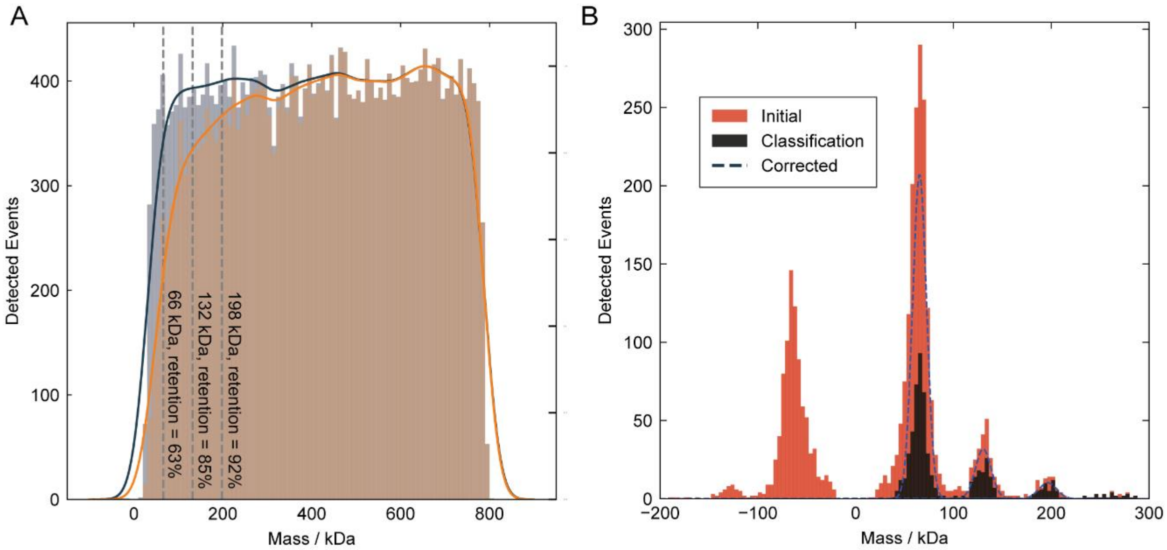

Fig. S4 – (A) Misclassification rates of optimal binding events across varying masses. Histogram of 29,901 simulated optimal events (slate blue) and retained events post-classification (orange). A normalised KDE plot overlays the histograms, used to calculate the retention rate of optimal events by mass. (B) Corrected BSA histogram, accounting for misclassification of optimal events.

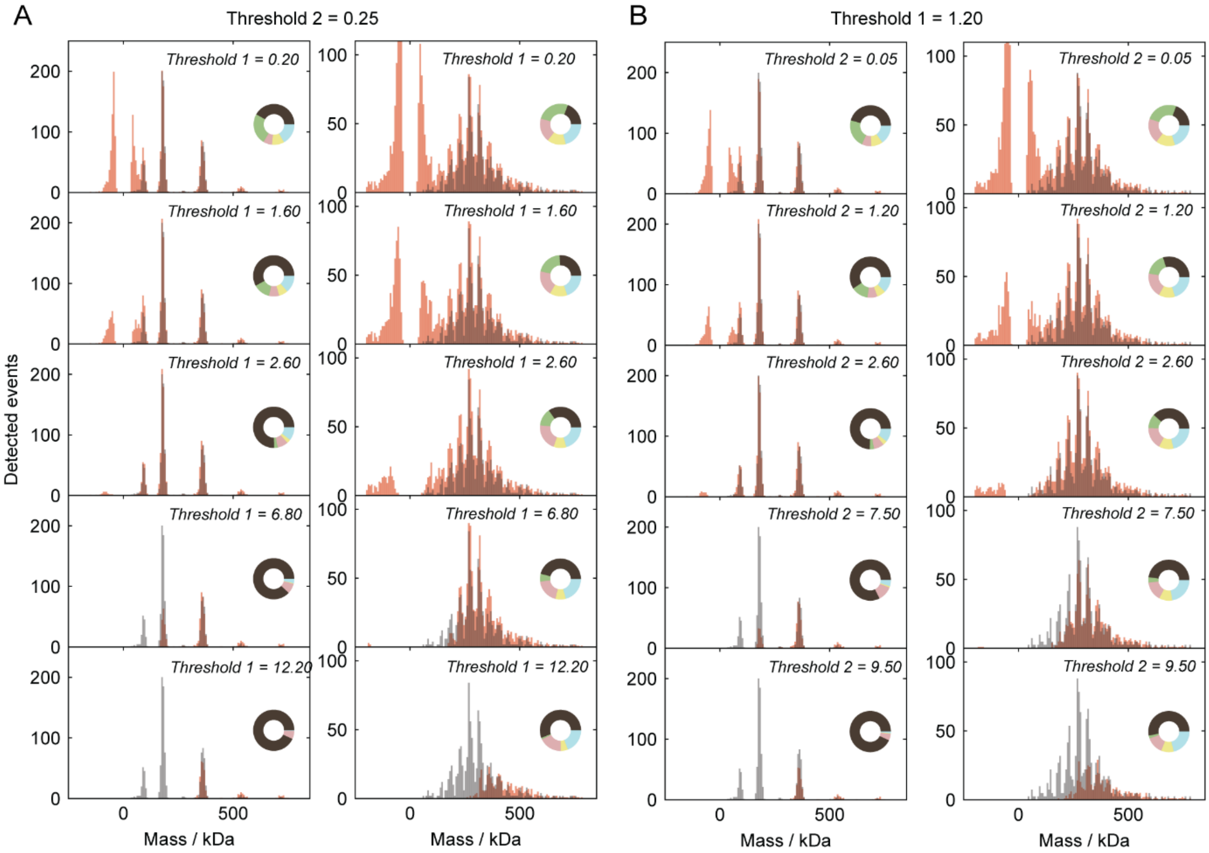

Fig S5 – The effect of adjusting Filter 1 and Filter 2 settings in Discover^{MP}. Demonstrated effects for Dynamin-ΔPRD and HSP27 bird samples, illustrating how arbitrary changes can alter the observed mass distribution. The Discover^{MP} analysis is shown in red, with the classification filtering analysis scaled and overlaid in grey. Pie chart insets display the distribution of event classes in the Discover^{MP}

analyses. (A) Effect of varying Filter 1 with Filter 2 fixed at 0.25. (B) Effect of varying Filter 2 with Filter 1 fixed at 1.20.

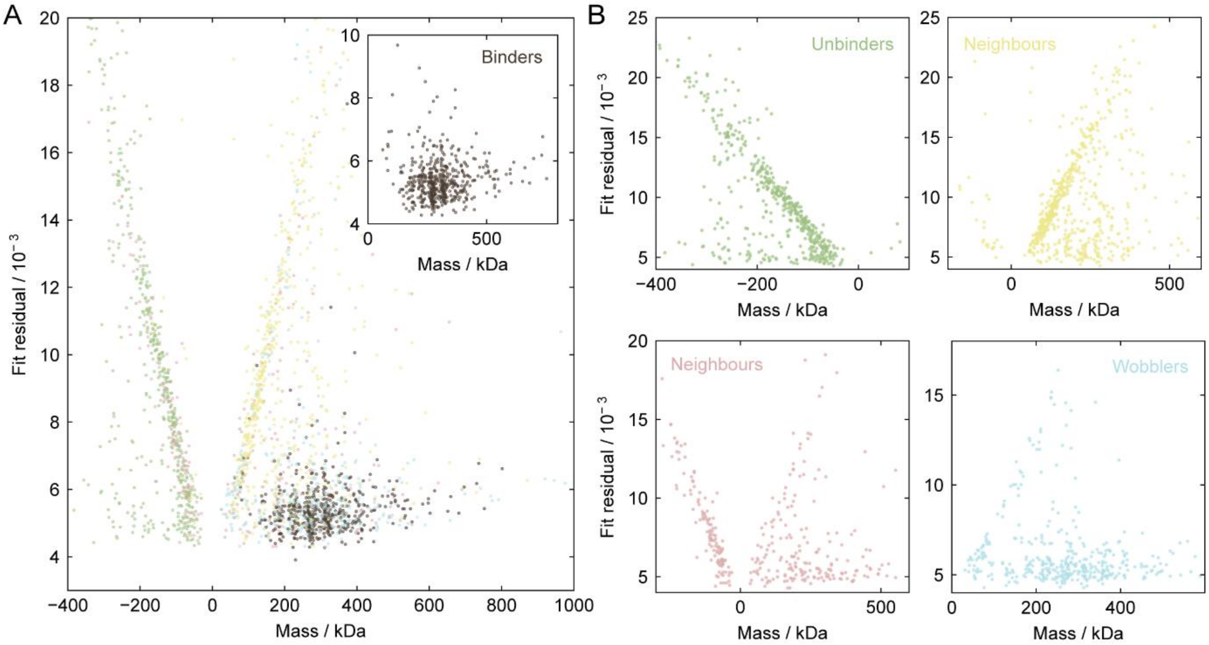

Fig S6– Contrast fit residuals for HSP27. (A) Combined fit residuals for all landing events, with an inset displaying the fit residuals for the optimal binder class. (B) Fit residuals separated for the suboptimal event classes.

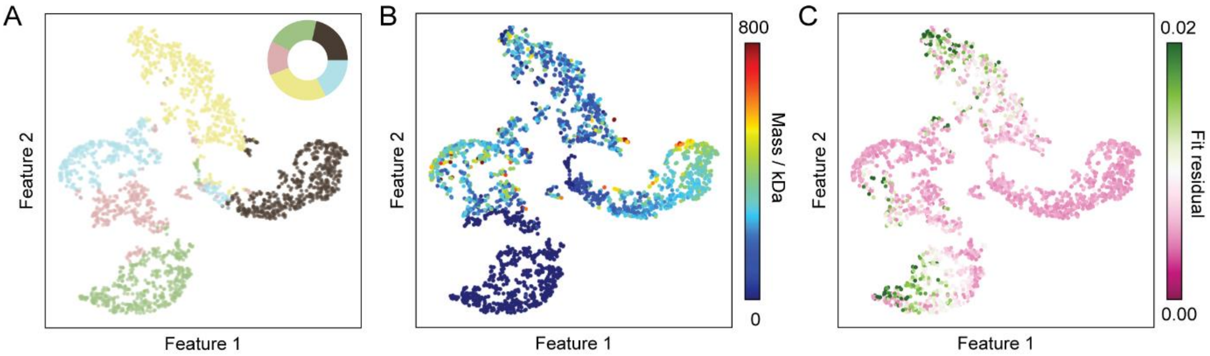

Fig S7 – t-SNE feature space representation for HSP27. (A) Event class distribution. (B) Mass distribution. (C) Fit residual distribution.

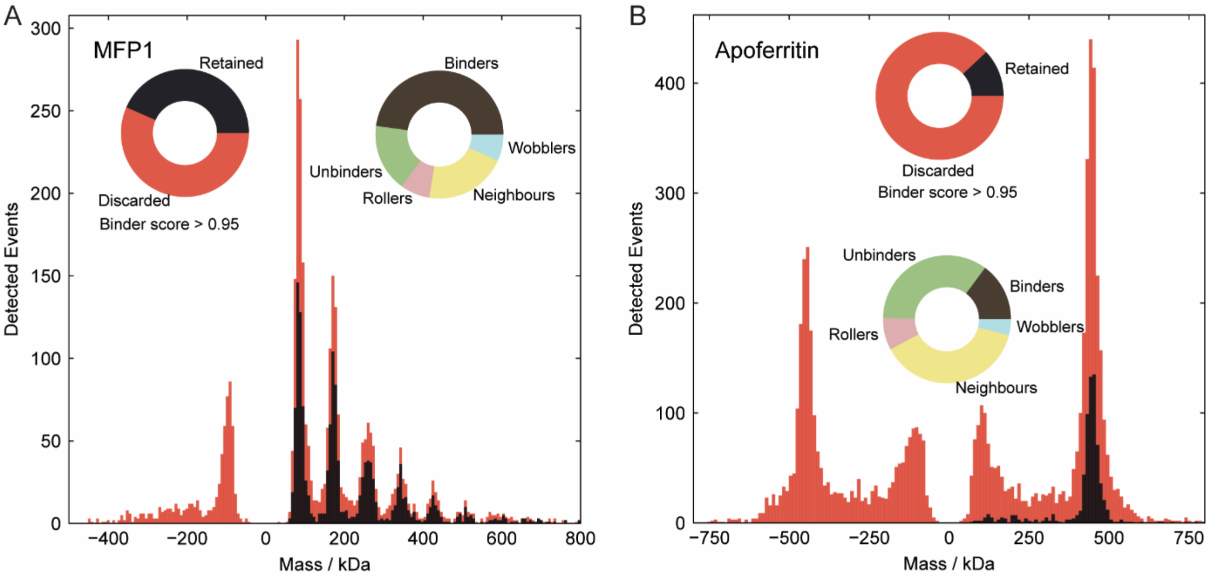

Fig S8 – Identifying optimal single-molecule landing events for massference-p1 and apoferritin.

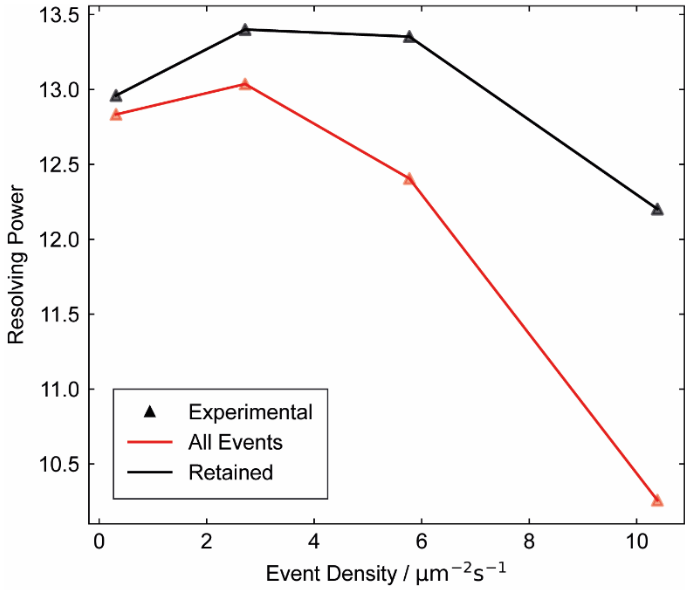

Fig S9 - Resolving power as a function of event density for the 360 kDa tetramer species, plotted before (red) and after (black) selective filtering. Experimental data.

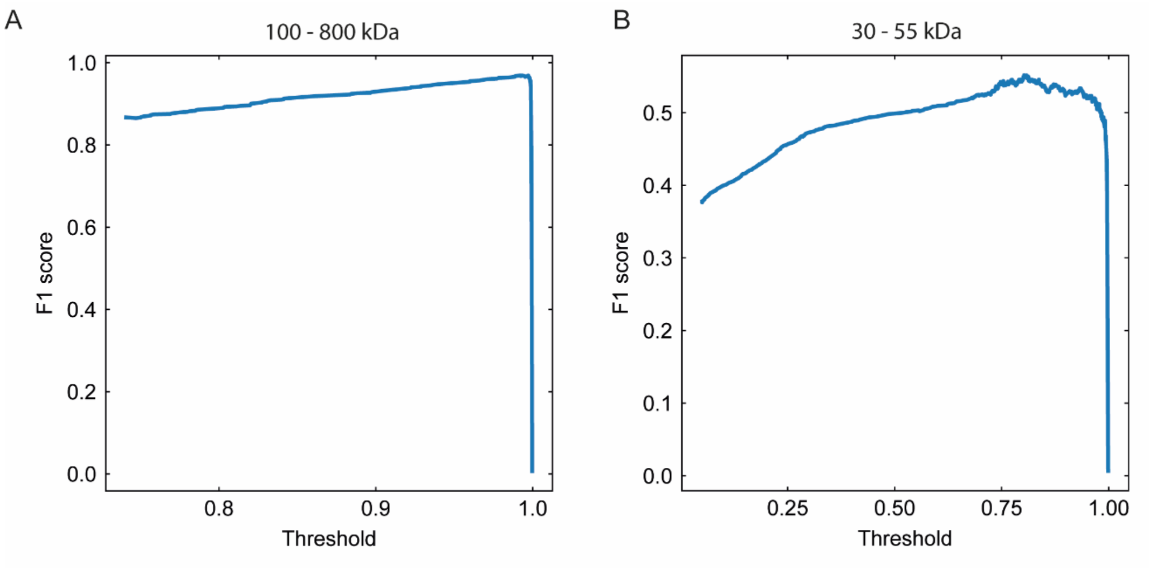

Fig S10 – F1 score as a function of binder-score threshold. (A) 100–800 kDa regime. (B) 30–55 kDa regime.

Supplementary Video 1 – Thumbnails of landing events from the HSP27 dataset, categorised into optimal and suboptimal events. Each thumbnail is individually z-scaled (colour bar) to optimise image contrast.
